# Recent Developments in Targeting Carbonic Anhydrase IX for Cancer Therapeutics

**DOI:** 10.18632/oncotarget.422

**Published:** 2012-01-28

**Authors:** Paul C. McDonald, Jean-Yves Winum, Claudiu T. Supuran, Shoukat Dedhar

**Affiliations:** ^1^ Department of Integrative Oncology, BC Cancer Research Centre and BC Cancer Agency, Vancouver, BC, Canada; ^2^ Institut des Biomolécules Max Mousseron (IBMM), UMR 5247 CNRS-UM1-UM2, Bâtiment de Recherche Max Mousseron, Ecole Nationale Supérieure de Chimie de Montpellier, 8 rue de l'Ecole Normale, 34296 Montpellier Cedex, France; ^3^ Laboritario di Chimica Bioinorganica, Universita degli Studi di Firenze, Italy; ^4^ Department of Biochemistry and Molecular Biology, University of British Columbia, Vancouver, Canada

**Keywords:** cancer, hypoxia, carbonic anhydrase IX, metastasis, targeted therapeutics

## Abstract

Carbonic anhydrase IX (CAIX) is a hypoxia-inducible enzyme that is overexpressed by cancer cells from many tumor types, and is a component of the pH regulatory system invoked by these cells to combat the deleterious effects of a high rate of glycolytic metabolism. CAIX functions to help produce and maintain an intracellular pH (pHi) favorable for tumor cell growth and survival, while at the same time participating in the generation of an increasingly acidic extracellular space, facilitating tumor cell invasiveness. Pharmacologic interference of CAIX catalytic activity using monoclonal antibodies or CAIX-specific small molecule inhibitors, consequently disrupting pH regulation by cancer cells, has been shown recently to impair primary tumor growth and metastasis. Many of these agents are in preclinical or clinical development and constitute a novel, targeted strategy for cancer therapy.

## INTRODUCTION

Hypoxia is a salient feature of many types of solid cancers and arises as the result of spatial disorganization and flow-based disruption of an abnormal microvasculature initiated by the growing tumor [1]. The impact of tumor hypoxia is multifaceted, with effects on several aspects of tumor biology, including genetic instability, angiogenesis, invasiveness, survival and metabolism [1, 2]. Reduced oxygen availability leads to the activation of a core cellular response to hypoxia, the master regulators of which are the transcription factors hypoxia-inducible factor 1 and 2 (HIF-1/2) [2]. Initiation of this core hypoxia-induced signaling cascade results in the activation of a vast array of genes, and from these arise signaling outputs that regulate a variety of processes aimed at adaptation of tumor cells to conditions of low oxygen [2]. Amongst the stressors imposed by hypoxia, the reduced supply of oxygen limits the capacity for oxidative phosphorylation as a means of producing energy [3, 4]. Hypoxic, HIF-1α-activated tumor cells respond to this microenvironmental stress by reprogramming their metabolism to engage the glycoytic pathway, a process that is far less efficient, but does not rely on the presence of oxygen. This “glycolytic switch” is often permanent and persists after reoxygenation, in part because the byproducts of glycolysis can be used for anabolic reactions that provide a selective advantage to highly proliferative tumor cells [3, 4]. The high rate of glycolysis maintained by cancer cells is the basis for the Warburg effect [3, 5].

The switch to glycolysis by highly metabolically active tumor cells results in increased production and export of acidic metabolites, such as lactic and carbonic acids, to the extracellular space and leads to a decline in extracellular pH (pHe) [6], creating a toxic intratumoral microenvironment and providing a selective advantage for tumor cells that can survive these harsh conditions. One consequence of extracellular acidification is the disruption of the intracellular pH (pHi), a decrease in which rapidly affects basic cellular functions, including membrane integrity, metabolism and energy production, and proliferation [4, 6]. Thus, cells must regulate pHi as the extracellular pH declines, a process particularly critical for tumor cells that prefer a pHi that is somewhat more alkyline compared to that which is optimal for normal cells [4, 7]. The combination of an increasingly acidic intratumoral microenvironment and a requirement to regulate pHi results in the death of non-tumor cells and accelerates degradation of the extracellular matrix, thereby promoting the invasion and proliferation of acid-resistant cancer cells. As such, metabolic alterations induced by hypoxia can promote activities associated with aggressive tumor cell behavior, including survival, invasion and metastasis [8].

The maintenance of pH homeostasis by tumor cells relies on a set of complex molecular mechanisms involving a variety of proteins and buffer systems with the central aim of maintaining a moderately alkaline pHi while generating a markedly acidic extracellular environment [4, 6]. One set of proteins important to this pH regulatory system is the family of carbonic anhydrases (CAs) [6, 9]. CAs are a family of 16 distinct, but related metalloenzymes whose major enzymatic function is to catalyze the reversible hydration of carbon dioxide (CO_2_) to bicarbonate (HCO_3_^−^ and protons (H^+^) (CO_2_ + H_2_O ↔ HCO_3_^−^ + H^+^) [9]. As a group, CAs are important regulators of a variety of biological processes, including respiration, acid-base regulation, bone resorption and calcification, and biosynthetic processes [9]. In the context of tumors, two particular isoforms, CAIX and CAXII, are associated with cancer progression, metastasis, and impaired therapeutic response [4].

## CAIX BIOCHEMICAL STRUCTURE

CAIX was initially identified as a membrane-bound protein on the surface of the HeLa human cervical carcinoma cell line and was named the “MN protein” [10, 11]. However, subsequent analysis of its cDNA sequence revealed the presence of a 257 aa long extracellular carbonic anhydrase (CA) domain, resulting in the acquisition of its current namesake [12, 13]. The recent solution of the structure of the catalytic domain of CAIX [14] has provided structural confirmation of its observed catalytic activity and has aided in more rational drug design [15-17]. In contrast to other CA isoforms, CAIX is dimeric [17], is among the most active CA for the CO_2_ hydration reaction [16] and contains a proteoglycan (PG)-like domain immediately adjacent to the catalytic domain, the presence of which may allow the enzyme to function most efficiently at more acidic pH values [17, 18]. A single pass transmembrane domain separates the extracellular portion of CAIX from a short, intracellular tail that is involved in regulating its enzymatic activity [19].

## REGULATION OF CAIX EXPRESSION

The transcriptional regulation of CAIX has been reviewed in detail recently [20] and the discussion here is limited its regulation as it pertains to cancer biology. Expression of CAIX by tumor cells is generally very low in normoxia, but levels are strongly induced during hypoxia [21-23]. The CAIX promoter contains a hypoxia responsive element (HRE) that is located immediately upstream of the transcription start site and that binds HIF-1α [21]. Indeed, HIF-1α is the exclusive regulator of CAIX activity, in contrast to many hypoxia-induced genes, and CAIX is often the most strongly upregulated gene in response to hypoxia in human cancer cells [9, 20]. Additional transcription factor binding sites present in the CAIX promoter appear to coordinate with the HRE to promote or amplify the HIF-1 response [20, 24].

Importantly, the signaling pathways, soluble factors, and microenvironmental conditions that regulate CAIX transcription converge on the HIF-1 pathway and CAIX expression is coupled directly both to increased protein stabilization and transcriptional activity of HIF-1 [20]. However, cancer cells grown to high density in vitro develop pericellular hypoxia and can upregulate the expression of CAIX as a consequence increased HIF-1 transcriptional activity, but in the absence of detectable stabilization of HIF-1, effectively “uncoupling” these two arms of HIF-1 regulation [20, 25, 26]. Culturing cancer cells at high density also induces signaling pathways, including the phosphatidylinositol 3-kinase (PI3K) pathway. Under these conditions, PI3K inhibitors suppress CAIX expression, revealing that CAIX is driven by increased transcriptional activity of HIF-1α resulting from mild hypoxia and PI3K activation [25, 27].

While hypoxia is the key driver of CAIX expression in cancer cells, there are also data suggesting that microenvironmental conditions, tumor suppressors and oncogenic signaling pathways may play a role in specific circumstances. In studies using glioblastoma cells grown in normoxia, acidification of the growth media was associated with increased CAIX promoter activity and upregulated CAIX expression, and was attributed to HIF-1 stabilization and activation of extracellular signal-regulated kinase (ERK) [28]. However, modulation of pHe had no effect on CAIX expression in normoxic HeLa cells [29], indicating that pH-driven upregulation of CAIX may be cell-type specific. The role of glucose deprivation has also been investigated, but results remain inconclusive, with some studies reporting heightened expression of hypoxia-induced CAIX in response to restricted glucose [29], while others have reported reduced CAIX expression in these conditions [30]. With regard to tumor suppressors, the best characterized example is the role of VHL in CAIX upregulation wherein mutation of VHL results in constitutive stabilization of HIF-1 in normoxia and drives hypoxia-independent expression of HIF-1 regulated genes, including CAIX [31]. Indeed, this mechanism results in the uniformly high CAIX expression reported in clear cell renal cell carcinoma [31]. P53 has also been shown to modulate CAIX expression, but again the primary target is HIF-1 [32]. Similarly, modulation of CAIX activity by the PI3K pathway and the ERK pathway have been reported, but the effects are cell-specific and the modulation of CAIX is likely secondary to the modulation of relevant transcription factors (HIF-1, SP1/SP3, AP1) [20, 25, 27].

## THE ROLE OF CAIX EXPRESSION IN NORMAL TISSUE

### CAIX expression in normal tissue

CAIX is an especially attractive target for cancer therapy, in part because it is overexpressed in a wide variety of solid tumors, but is expressed in a limited way in normal tissues. Early studies established that the differential distribution in cancerous versus normal tissue is not the result of mutations [33]. In human tissue, strong expression of CAIX is generally limited to the basolateral surface of proliferating crypt enterocytes of the duodenum, jejunem and ileal mucosa in the human [34]. However, diffuse, weak CA IX expression has also been reported in the epithelia of human male efferent ducts [35], as well as in occasional foci in the pancreatic acini [36]. Analyses based on cDNA demonstrated the presence of CA IX in gall bladder epithelia [33], but protein has not been detected.

In mice, CAIX expression is evident by days E11.5-E12.5 [37, 38] and is expressed to significant levels in the brain, lung, liver and pancreas, while weak signals are evident in the kidney and stomach [38]. Expression in the normal adult is restricted to a few tissues, with the gastric mucosa exhibiting the highest levels of protein [37]. Post transcriptional control of CAIX may be evident in some tissues as relatively strong signals were observed for CAIX mRNA in the kidney and skeletal muscle of Balb/c mice, but these tissues showed little or no expression of the protein [37]. Indeed, normal human kidney and muscle are essentially null for CAIX mRNA and protein [39]. Mice also show expression of CAIX in pancreatic acini in a diffusely distributed pattern [37].

### Phenotypes in CAIX knock-out animals

As would be predicted from the observations of restricted expression of CAIX in normal adult tissues, genetic disruption of CAIX expression in mice results in mild phenotypes. Global knockout of CAIX in mice by targeted gene disruption resulted in homozygous animals developing gastric hyperplasia of the glandular epithelium, including numerous cysts [40]. The hyperplasia was prominent within 4 weeks after birth and loss of CA IX expression led to increased numbers of pit cells and depletion of pepsinogen-secreting chief cells [40]. However, mice developed normally and showed normal gastric pH, acid secretion and serum gastrin levels [40]. No effect on the hyperplasia and no dysplasia were noted when knockout animals were challenged with a high salt diet [41], although gastric submucosal inflammation was noted in one mouse strain [41]. Importantly, CAIX deficiency did not promote tumorgenicity [41], although aged CAIX null mice may suffer degenerative disease in the brain. At 8 to 10 months of age, vacuolar degenerative morphological changes were noted in knockout animals [42]. Interestingly, CAIX expression is upregulated about 2 –fold in mice null for CAII [43], suggesting the presence of functional redundancy amongst the CA isozymes.

## CAIX EXPRESSION IN TUMOR TISSUE

The discussion above reinforces the fact that CAIX expression in the normal adult is highly restricted and that interference with its function in normal tissue likely has few, if any, significant consequences, except perhaps in chronic, age-related situations. On the other hand, CAIX is overexpressed in many solid tumors and there is now a well-established relationship between the expression of CAIX and patient prognosis. CAIX expression, as detected by immunohistochemical staining of tissue sections, is upregulated and associated with poor prognosis in cancers of the lung [44-46], colon [47, 48], breast [49-51], cervix [52-55], bladder [56], ovaries [57], brain [58], head and neck [59-61], and oral cavity [62, 63]. Furthermore, recent studies have examined the expression of CAIX in cohorts of hundreds to thousands of patients using tissue microarray (TMA) strategies (Table 1). Using this high throughput platform, CAIX has been validated as a biomarker of poor prognosis in breast [23, 64], lung [46], ovarian [57] and bladder cancer [65], as well as in astrocytomas [66].

**Table 1:** Tissue microarray studies evaluating CAIX as a poor prognostic biomarker in human cancer

Cancer Type [citation]	# of Samples	Total CAIX +ve (%)	Prognostic Indicator					
			Univariate Analysis			Multivariate Analysis		
			DSS	OS	MFS	DSS	OS	MFS
**Breast [23]**	3630	16	Yes	NR	Yes	Yes	Yes	Yes
**Breast [64]**	144	26	NR	Yes	NR	NR	Yes	NR
**NSCLC [46]**	555	24	Yes	Yes	NR	Yes	NS	NR
**Ovarian [57]**	205	26	NR	Yes	NR	NR	Yes	NR
**Bladder [65]**	340	71	NR	Yes	NR	NR	Yes	NR
**Astrocytoma [66]**	362	78	NR	Yes	NR	NR	Yes	NR

DSS, disease specific survival; OS, overall survival; MFS, metastasis free survival; NR, not reported; NS, not significant.

While the vast majority of studies support a correlation between CAIX overexpression and patient prognosis, a few studies have failed to find a relationship. For example, in a TMA study of 99 patients that encompassed pre and post (chemo)radiotherapy biopsies, CAIX was detected in over 85% of pre-treatment specimens [67]. However, the study failed to find a relationship with prognosis, probably because of insufficient numbers of patients [67]. On the other hand, in a series of 166 patients with rectal cancer where full tissue sections were used, 44% were positive for CAIX expression and two thirds of these patients showed moderate to strong staining [48]. High CAIX expression was indicative of shorter disease-free and disease specific survival, and was demonstrated to be an independent poor prognostic biomarker [48]. Similarly, most clinical studies have demonstrated that a high level of CAIX is a biomarker of poor prognosis both in local advanced [52-55] and in early stage [68, 69] cervical cancer, but some studies have failed to find a correlation [70].

In addition to overall prognostic information, interrogation of TMAs has revealed associations between CAIX overexpression and specific tumor categories. For example, CAIX is particularly highly expressed in basal-like or triple negative breast cancers, a tumor subgroup with poor prognosis and known resistance to therapy [23, 51]. Tumor type dependence has also been reported in lung cancer, with a higher percentage of CAIX positive tumors amongst the squamous cell phenotype [46]. Clinical studies have also revealed a correlation between CAIX expression and metastatic disease. Most recently, interrogation of over 3600 human breast cancers provided definitive evidence of CAIX as an independent biomarker of poor prognosis for distant metastases [23]. CAIX expression was interrogated in a TMA series of 930 breast cancers categorized according to patient survival and overexpression was correlated with death and metastatic disease [71]. Several studies have now demonstrated that CAIX levels are associated with metastasis-free survival in multivariate analyses [52-54, 69], and high levels of CAIX expression are associated with greater lymph node metastases [54, 55].

There is now increasing focus on the use of soluble, plasma CAIX for clinical detection and prognostic evaluation. Initial studies involving relatively small sample sizes showed that soluble, serum CAIX was upregulated in patients with solid tumors [72, 73] and could be used to detect clinically relevant disease, although prognostic impact was not realized. However, recent studies have shown an association between soluble CAIX and patient prognosis. Preoperative serum CAIX concentrations in vulvar cancer correlated with intratumoral expression and increased serum CAIX levels were associated with poor prognosis [74]. Serum levels of CAIX in metastatic breast cancer were also correlated with poor prognosis, as well as with the incidence of circulating tumor cells [75]. Similarly, in NSCLC, high plasma levels of CAIX were associated with significantly shorter overall survival [46].

## BIOLOGICAL FUNCTIONS OF CAIX IN CANCER CELLS

### CAIX and control of tumor pH

**Figure 1:**
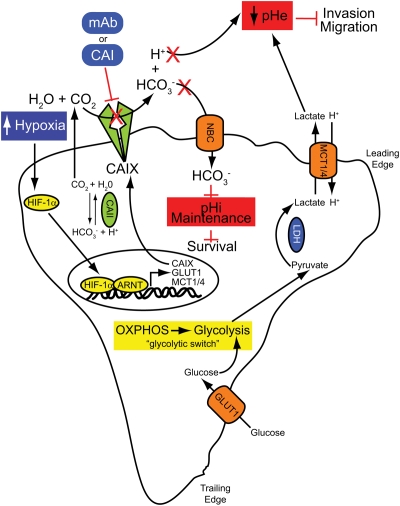
Pharmacologic inhibition of CAIX activity inhibits cancer cell survival and invasion Hypoxia induces a HIF-1-mediated signaling cascade that results nuclear translocation of HIF-1α and activation of hypoxia-regulated genes, including GLUT1, MCT1/4 and CAIX. Cells reprogram their metabolism to engage the glycoytic pathway, a “glycolytic switch” that results in increased production and export of lactate, leading to a decline in pHe. One consequence of extracellular acidification is the disruption of the intracellular pH (pHi), a decrease in which rapidly affects basic cellular functions. The overexpression of CAIX in hypoxia catalyzes the hydrolysis of CO_2_ to HCO_3_^−^ and H^+^ in the extracellular microenvironment. The HCO_3_^−^ is actively transported into the cancer cell, thereby regulating pHi and maintaining cell survival. The H^+^ participate in the generation of an increasingly acidic extracellular space, promoting tumor cell invasiveness. Inhibition of CAIX catalytic activity using monoclonal antibodies or specific small molecule inhibitors prevent the production of these enzymatic metabolites, leading to decreased survival and reduced invasive capacity.

The role of CAIX in the control of pH dynamics in solid tumors is now well-recognized [6, 76]. The maintenance of pH homeostasis by tumor cells encompasses several complex molecular mechanisms involving a variety of proteins and buffer systems with the central aim of maintaining a moderately alkaline pHi while generating a markedly acidic extracellular environment [6] (Figure 1). Indeed, tumor cells generally favor a more alkaline pHi than their normal counterparts [4]. A change in the pHi/pHe ratio of 0.1-0.2 pH units can have disasterous consequences for critical biological processes including ATP synthesis, proliferation, migration, survival and metastasis [6, 8]. The primary enzymatic function of CAIX is to catalyze the reversible hydration of carbon dioxide to bicarbonate and protons (CO_2_ + H_2_O ↔ HCO_3_^−^ + H^+^). The location of the active site of CAIX on the extracellular surface of the plasma membrane positions it well for the enzyme to contribute to acidification of the tumor microenvironment in hypoxia. In fact, CAIX has been said to act as a “catalytic converter” for the excretion of acid from cells [77]. Bicarbonate is shuttled into the cytoplasm to buffer pHi, while the proton remains in the extracellular space. Thus, CAIX has a dual role in the growth of hypoxic, CO_2_ excreting tumors (Figure 1). First, it helps to produce and maintain an alkyline pHi favorable for tumor growth. Second, it participates in the generation of an increasingly acidic extracellular space, facilitating tumor cell invasiveness [6, 76, 77]. Inhibition of CAIX interferes with removal of acid and results in a decrease in pHi, negatively influencing cell survival [76, 77] (Figure 1). Furthermore, low pHe is associated with tumorigenic transformation, chromosomal rearrangements, extracellular matrix breakdown, migration and invasion, protease activation and growth factor production [6, 76]. Inhibition of CAIX would also attenuate these processes, thereby reducing tumor growth and invasion (Figure 1).

### Regulation of CAIX activity

Recent data have suggested that the regions flanking the catalytic domain, in particular the PG-like domain and the intracellular tail, are critical for modulating the catalytic activity of CAIX. The PG-like domain allows CAIX to remain highly catalytically active at low pH values that would kill most enzymes [17]. Mutagenesis of a cluster of basic amino acids in the intracellular tail affected the function of the extracellular catalytic domain, suggesting that the cytoplasmic tail plays a role in inside-out signaling [78, 79]. In addition, the intracellular tail contains three potential phosphorylation sites, ^443^T, ^448^S and ^449^Y. Data suggest that ^449^Y plays a role in EGF-R-induced signaling to Akt [80], while cAMP-mediated activation of PKA in hypoxia leads to PKA-induced phosphorylation on ^443^T of CAIX, resulting in increased activity [19]. Interestingly, full activation of CAIX also appears to require dephosphorylation of ^448^S [19].

### CAIX in cell adhesion, migration and proliferation

In addition to its role in the regulation of tumoral pH and tumor cell survival, there is evidence that CAIX contributes to cell processes such as adhesion and migration, both of which are vital for metastatic progression in human cancer. In CAIX-null MDCK cells constitutively expressing human CAIX, the protein localized to cell-cell junctions and overlapped with staining for E-cadherin [81]. Reoxygenation of hypoxia-cultured cells resulted in delocalization of CA IX and E-cadherin from the membrane to the cytoplasm, followed by relocalization to the membrane upon return to hypoxia [81]. CAIX-expressing MDCK cells showed lower cell adhesion and CA IX immunoprecipitated with β-catenin in these cells, suggesting that CAIX may modulate E-cadherin-mediated cell adhesion by competing with E-cadherin for binding to β-catenin [81]. Gene expression analysis of cervical carcinoma cells overexpressing exogenous human CAIX revealed differential regulation of genes involved in cell growth, cell adhesion and cytoskeletal organization [82]. These cells showed weakened cell-cell adhesion and increased cell motility. Moreover, constitutive CAIX expression induced inactivation of Rho and led to EMT in these cells [82]. The expression of CAIX may also be involved in cell proliferation. Several studies have also reported a reduction in proliferation when CAIX is inhibited, at least in cells exogeneously overexpressing CAIX [83-85]. Together, these studies suggest that inhibition of CAIX in hypoxic tumors may have anti-tumor effects that extend beyond control of intratumoral pH.

## PHARMACOLOGIC INHIBITION OF CAIX AS A MEANS OF THERAPEUTIC INTERVENTION IN CANCER

CAIX is an attractive target for anticancer therapy for several reasons. It is selectively expressed by tumor cells and shows highly restricted expression in normal tissue. It provides functions critical for tumor growth and metastasis, including pH regulation, survival and adhesion/migration. It is positioned on the extracellular surface of cell membranes, allowing efficient targeting by antibodies or small molecule inhibitors. Furthermore, genetic silencing of CAIX in preclinical tumor models in vivo has demonstrated the requirement of CAIX for the growth of hypoxic tumors and their metastasis. Genetic depletion of CAIX, together with CAXII, in LS147Tr colorectal cancer xenografts resulted in an 85% reduction in tumor growth [86]. Silencing of CAIX expression in 4T1 mouse metastatic breast cancer cells resulted in regression of orthotopic mammary tumors and inhibition of spontaneous lung metastasis formation [23]. Stable depletion of CAIX in MDA-MB-231 human breast cancer xenografts also resulted in attenuation of primary tumor growth [23]. These studies provide proof of principle data showing that inhibition of CAIX is of potential therapeutic benefit. Two major tools that are being investigated for their efficacy as CAIX-specific therapeutic modalities in cancer treatment are monoclonal antibodies and small molecule inhibitors.

### Monoclonal antibodies

Immunotherapy using CAIX-specific monoclonal antibodies (mAbs) may derive its therapeutic efficacy through a variety of mechanisms. Direct binding of the mAb to CAIX can elicit an anti-tumor response due to antibody-mediated cell cytotoxicity (ADCC). Alternatively, receptor-mediated internalization allows for targeted delivery of various therapeutic payloads, including cytotoxins and radionuclides. Furthermore, development of mAbs targeting the CAIX active site provides potential for selective blocking of CAIX function.

Historically, two mAbs, M75 [10] and G250 [87], have dominated as CAIX-specific immunological tools for clinical detection and/or therapy. M75 is a highly-specific antibody targeting the PG-like domain of CAIX and it is used widely for immunohistochemical detection of CAIX in human tumor tissue [79]. A radiolabeled derivative has been developed for imaging CAIX in hypoxic tumors preclinically [88, 89], but M75 has not been developed for application as an immunotherapy. In contrast, a chimeric version of G250, cG250, has been developed and extensively characterized as an anticancer immunotherapy [90]. Early studies showed that cG250 could elicit antibody-dependent cellular cytotoxicity (ADCC) [91], an established mechanism by which therapeutic mAbs mediate tumor cell destruction. Phase I and II trials demonstrated that this Ab was safe, well-tolerated and able to positively impact disease burden, alone and together with interferon (IFN)-α treatment [92, 93]. cG250 is currently marketed by WILEX AG under the trade name RENCAREX® and phase III trials have now been initiated using cG250 as an adjuvant therapy aimed at reducing recurrence in surgically-treated renal cell carcinoma (RCC) patients who have a high risk of relapse and in combination with interleukin 2 (IL-2) or IFN-α for metastatic RCC [4, 79, 92].

While M75 and cG250 are directed at the PG-like domain of CAIX, recent studies have focused on developing antibodies targeted against its catalytic domain. These antibodies have the advantage of specifically disrupting the catalytic activity of the enzyme, thus targeting its tumorigenic functions, including pH regulation. High throughput screening technologies, especially the use of phage display libraries [94-96], have been employed to efficiently identify CAIX antibodies. Epitopes of some of the fragments have mapped to the catalytic site, and these have been shown to inhibit CAIX activity in vitro [94, 96]. Inhibition of CAIX in spheroid cell cultures has also been demonstrated, suggesting that these mAbs could be used to inhibit CAIX therapeutically [94]. However, in vivo experiments demonstrating efficacy as anti-tumor agents have not been performed. Two mAbs generated by phage display and with high affinity to CAIX have been shown to bind CAIX in tumors in vivo [95], but again, evaluation of anti-tumor efficacy was not performed. On the other hand, a mAb directed against the CAIX catalytic domain and generated using hybridoma technology has been developed and tested in vivo [79]. The mAb showed efficient binding, internalization and persistence in cultured cells. In vivo evaluation in HT-29 colorectal xenografts demonstrated that treatment immediately after cell inoculation effectively limited tumor growth, but the effect was very modest when tumors were allowed to establish prior to treatment [79].

While selective inhibition of CAIX catalytic activity by mAbs exploits the property of mAbs to not spontaneously cross the plasma membrane, the utility of mAbs in cancer therapy is often driven by their ability to undergo receptor-mediated internalization. Ab internalization is required for delivery of therapeutic payloads, including radionuclides and cytotoxic drugs, and CAIX Abs have been developed to exploit this property. For example, a portion of the cG250 Ab is internalized, and metallic radionuclides, including ^177^Lu and ^90^Y, have been introduced into cellular lysosomes using cG250 in mice, producing a therapeutic growth delay in xenograft tumors [97]. A phase I/II trial in metastatic RCC patients is currently ongoing [98]. In a very recent study a CAIX mAb identified from a phage display library was conjugated to monomethyl auristatin E (MMAE) and the efficacy of this Ab-drug conjugate (BAY79-4620) was evaluated in several preclinical human xenograft tumor models [99]. Drug delivery to target cells involved internalization of the mAb and anti-tumor efficacy correlated with CAIX expression, with tumors showing the most robust levels of CAIX expression also having the best therapeutic response [99].

### CAIX-specific small molecule inhibitors

Several broad classes of small molecules are known to effectively inhibit CAs [4, 9], and compounds based on sulfonamide/sulfamates and coumarins have demonstrated particular promise as potential anti cancer agents. Interestingly, these two inhibitor classes are mechanistically distinct in their inhibition of CAs. The sulfonamides inhibit CAIX by coordinating to the zinc ion within the active site, while molecules based on coumarin/thiocoumarin act as suicide inhibitors, undergoing hydrolysis to 2-hydroxycinnamic acids and binding irreversibly at the entrance to the active site cavity [100, 101].

### Evaluation of CAIX inhibitors in cell culture

The high degree of homology amongst the catalytic sites of the various mammalian CAs has presented a challenge to the design and development of isozyme-specific agents. The development of compounds that selectively inhibit tumor associated, extracellular CAs without “off-target” inhibition of intracellular CAs such as CAII is critical for their use as cancer therapeutics [4, 9]. Several strategies have evolved to “dial in” the selectivity of small molecule inhibitors of CAIX, such as the addition of charges species, bulky entities such as FITC or albumin or hydrophilic sugar moieties, all of which limit transport across the plasma membrane [4, 9]. Other approaches include the use of distinct chemotypes of sulfonamides [102] or coumarins [103] that are inherently selective for extracellular CAs such as CAIX. Many of these compounds have been evaluated as both potent and selective for CAIX based on in vitro assays using recombinant CAs. In some studies, efficient attenuation of cell growth by a series of chemically defined CA inhibitors has been demonstrated using panels of cultured tumor cell lines representing a variety of types of cancers [104, 105]. While these studies have identified CA inhibitors as potential anti-cancer therapeutics, the experiments were performed in normoxia without analysis of CAIX status, thus evaluation of the specific role of CAIX on tumor cell viability was not possible. Indeed, these studies suggest that some of these CA inhibitors may work effectively as cytotoxic agents in cancer therapy [104, 105]. Ideally, inhibitors that specifically target CAIX-overexpressing cancer cells while exhibiting no effects on normal, CAIX-negative cells would be desired.

A growing number of selective CAIX inhibitors have been evaluated in cell culture models and have provided evidence both of their CAIX-specific nature and their potential as anti-tumor agents. To date, the best characterized CAIX-selective small molecule inhibitor is a FITC-labeled fluorescent sulfonamide [9]. This compound has a high affinity for extracellular CAs. Cell culture studies using both MDCK cells that overexpress CAIX and human tumor cell lines that constitutively express CAIX or induce the enzyme in hypoxia have demonstrated that this inhibitor binds only to active CAIX in hypoxia [23, 106] and inhibits CAIX-mediated acidification of the extracellular environment [23, 106, 107]. Interestingly, similar data was reported recently for a fluorescently labeled sulfamate [84]. The influence of CAIX inhibitors on cancer cell viability has also been investigated. Human renal carcinoma cell lines with or without constitutive expression of CAIX, together with cell density dependent upregulation of CAIX in HeLa cells, were used as models to test the functional impact of two aromatic sulfonamides, TR1 and GA15 [83]. Results showed that these inhibitors preferentially reduced pHi and proliferation, and induced apoptosis and ceramide production in CAIX-positive cells, but not in CAIX-negative cells [83]. Treatment with an indanesulfonamide CAIX inhibitor in HT-29 human colon carcinoma cells resulted in reduced proliferation and increased apoptosis, but did not affect intrinsic radiosensitivity in this cell line [84]. Such studies demonstrate that sulfonamide inhibitors can be engineered to bind selectively to CAIX and inhibit its biological functions in cell culture. Interestingly, some of these experiments were carried out in normoxia [83], suggesting that certain CAIX inhibitors may function regardless of hypoxia-induced activation. Thus, these inhibitors can negatively impact cancer cell viability in a CAIX-dependent manner.

### Evaluation of selective CAIX inhibitors in preclinical models

Despite great scientific interest in CAIX as a biomarker, imaging target and therapeutic target for hypoxic tumors, preclinical evaluation of targeted agents remains in its infancy. The paucity of appropriate, CAIX-positive preclinical models and the complexities involved in synthesizing small molecule inhibitors that are highly selective for the CAIX isozyme remain as challenges in the field [4, 23, 108]. Recently, however, progress has been made in developing small molecule inhibitors with reasonable selectivity for extracellular CAIX that demonstrate efficacy in vivo (Table 2).

**Table 2:** Targeting of CAIX by small molecules in preclinical models of human cancer

Tumor Model [citation]	Inhibitor	Effect
LS174T human colon carcinoma [109]	fluoro-acetazolamide	bound to xenograft tissue; no significant effect on tumor growth
SK-RC-52 human renal cell carcinoma [109]	albumin-acetazolamide	significant reduction in tumor growth
HT-29 human renal cell carcinoma [110]	bis sulfonamide	tumor-specific accumulation; effect on tumor growth not measured
HT-29 human renal cell carcinoma [111]	fluorescent sulfonamide	tumor-specific accumulation; effect on tumor growth not measured
4T1 metastatic mouse breast cancer [23]	fluorescent sulfonamide	significant inhibition of tumor growth
4T1 metastatic mouse breast cancer [23, 102]	ureido sulfonamides	inhibition of experimental lung metastases
4T1 metastatic mouse breast cancer [23, 103]	glycosyl coumarins	inhibition of experimental lung metastases; inhibition of primary tumor growth
MDA-MB-231 LM2-4 lung metastatic breast cancer [23]	ureido sulfonamides	inhibition of primary tumor growth
HT-29 human renal cell carcinoma [84]	indanesulfonamide	inhibition of primary tumor growth; synergy with radiotherapy

Initial attempts to target CAIX therapeutically in vivo used inhibitors based on acetazolamide, onto which albumin binding moieties or charged flurophores were attached, thereby restricting the binding of acetazolamide to extracellular CAs [109]. The fluorophore-conjugated inhibitor bound to LS174T human colon carcinoma xenograft tissue in a pattern similar to that seen with antibodies against CAIX, but a decrease in tumor growth was not observed in this model [109]. However, treatment of SK-RC-52 human renal cell carcinoma xenografts constitutively expressing CAIX with the albumin-binding variant did produce a significant decrease in tumor growth [109]. The inhibitor was also effective when used in combination with sunitinib, although the combination treatment did not significantly outperform sunitinib alone [109], likely because of the high efficacy of sunitinib in this tumor model. More recently, one of several potent bis-sulfonamide CAIX inhibitors identified in a screen of 1 million compounds in a DNA-encoded chemical library was found to exhibit strong tumor-specific accumulation in these tumor models [110]. Tumor-specific, oxygen-dependent localization of the established fluorescent sulfonamide inhibitor of CAIX in HT-29 colon carcinoma xenografts has also been reported [111]. While tumor growth inhibition per se was not measured in these studies, the results indicate the ability of highly selective CAIX inhibitors to bind specifically to their target in vivo.

Recent studies have now demonstrated that CAIX-selective sulfonamide inhibitors can directly and specifically inhibit the growth of hypoxic, CAIX-positive tumors in preclinical animal models. Treatment of hypoxic, metastatic 4T1 mouse breast tumors in an orthotopic setting with an established fluorescent sulfonamide CAIX inhibitor resulted in significant inhibition of tumor growth, whereas similar treatment of primary tumors derived from non-metastatic 67NR cells did not have an effect on tumor growth [23]. Furthermore, delivery of novel ureido sulfonamide [102] and glycosyl coumarin [103] inhibitors of CAIX into human and mouse models of orthotopic, CAIX-positive breast cancer resulted in significant inhibition of primary tumor growth. An anti-tumor effect has also been demonstrated recently by inhibiting CAIX using an indanesulfonamide [84]. Interestingly, treatment of HT-29 xenografts with this high affinity inhibitor of CAIX resulted in reduced tumor growth, and further regression what observed when the inhibitor was used in combination with radiotherapy [84]. Collectively, these studies provide strong preclinical evidence for the use of CAIX inhibitors as targeted pharmacologic agents for the treatment of hypoxic, aggressive solid cancers.

In addition to their effects on the growth of hypoxic primary tumors, recent data suggest that sulfonamide and coumarin inhibitors of CAIX activity are efficacious in reducing metastatic burden in preclinical models of cancer. In two separate studies, treatment of experimental models of breast cancer metastasis with novel ureido sulfonamides has resulted in a significant decrease in lung metastases [23, 102]. Similar results have been achieved using novel glycosyl coumarins [23, 103], suggesting that this new generation of selective CAIX inhibitors have the capacity to work as targeted therapeutics for both the treatment of primary tumors and metastatic progression, at least in breast cancer.

## CONCLUSION AND PERSPECTIVES

As solid malignancies progress, they often become hypoxic, triggering a core, HIF-1-mediated signaling cascade that results in the activation of a plethora of genes vital for the adaptation of tumor cells to an increasingly stressful environment. As part of this response, tumor cells undergo a glycolyic switch that enables them to continue to grow in conditions of low oxygen. As a consequence of this altered metabolism, acidic metabolites are released into the extracellular environment, lowering the pHe and putting pressure on tumor cells to maintain their pHi. Therefore, cancer cells become increasingly reliant on a multi-component pH regulatory system for their continued survival and invasive behavior, and interference with one or more effectors of this system can prove detrimental to tumor growth.

CAIX is a hypoxia-inducible component of the tumoral pH regulatory system. Its upregulation results in dramatically enhanced hydrolysis of CO_2_ to HCO_3_^−^ and H^+^ at the extracellular surface of cancer cells, providing a chemical buffer that is actively transported into the cell to help maintain pHi and a source of H^+^ that contributes to the increasing acidosis of the extracellular space. As discussed here, it is now well-recognized that CAIX is highly overexpressed in several solid cancer types, in sharp contrast to the restricted expression of this protein in normal tissues, making it a clinically relevant target for novel cancer therapeutics.

Preclinical and clinical evaluation of novel CAIX-selective therapies have, until recently, been slowed by the lack of availability of appropriate animal models and the development of chemical compounds that selectively inhibit tumor associated, extracellular CAs without “off-target” inhibition of intracellular CAs. However, several CAIX-targeted therapeutic agents, including monoclonal antibodies and small molecule inhibitors, have been developed recently. Today, selective monoclonal antibody therapies have entered clinical trials and several additional antibody-based modalities show promise in experimental models. Importantly, inhibition of CAIX with sulfonamide- or coumarin-based inhibitors is efficacious in reducing tumor growth and inhibiting metastasis in preclinical tumor models without the compromising effects of non-specific toxicity. These attributes suggest that such inhibitors are likely to be useful clinically, especially if administered in combination with conventional chemotherapy. Indeed, the use of nontoxic targeted therapies in combination with conventional anticancer drugs or radiotherapy is the current clinical paradigm, and the inhibition of CAIX in this context may yield even better efficacy.
